# Reliability and Usability Analysis of an Embedded System Capable of Evaluating Balance in Elderly Populations Based on a Modified Wii Balance Board

**DOI:** 10.3390/ijerph191711026

**Published:** 2022-09-03

**Authors:** Ángel Gabriel Estévez-Pedraza, Enrique Hernandez-Laredo, María Elena Millan-Guadarrama, Rigoberto Martínez-Méndez, María Fernanda Carrillo-Vega, Lorena Parra-Rodríguez

**Affiliations:** 1 Faculty of Medicine, Universidad Autónoma del Estado de México, Toluca de Lerdo 50180, Mexico; 2 Faculty of Engineering, Universidad Autónoma del Estado de México, Toluca de Lerdo 50100, Mexico; 3 Faculty of Nursing and Obstetrics, Universidad Autónoma del Estado de México, Toluca de Lerdo 50180, Mexico; 4 Research Department, Instituto Nacional de Geriatría, Mexico City 10200, Mexico

**Keywords:** balance assessment, center of pressure (CoP), modified Wii Balance Board (mWBB), reliability, older adults

## Abstract

This paper analyzes the reliability and usability of a portable electronic instrument that measures balance and balance impairment in older adults. The center of pressure (CoP) metrics are measured with a modified Wii Balance Board (mWBB) platform. In the intra- and inter-rater testing, 16 and 43 volunteers (mean 75.66 and standard deviation (SD) of 7.86 years and 72.61 (SD 7.86) years, respectively) collaborated. Five volunteer raters (5.1 (SD 3.69) years of experience) answered the System Usability Scale (SUS). The most reliable CoP index in the intra-examiner tests was the 95% power frequency in the medial-lateral displacement of the CoP with closed-eyes. It had excellent reliability with an intraclass correlation coefficient ICC = 0.948 (C.I. 0.862–0.982) and a Pearson’s correlation coefficient PCC = 0.966 (*p* < 0.001). The best index for the inter-rater reliability was the centroidal frequency in the anterior-posterior direction closed-eyes, which had an ICC (2,1) = 0.825. The mWBB also obtained a high usability score. These results support the mWBB as a reliable complementary tool for measuring balance in older adults. Additionally, it does not have the limitations of laboratory-grade systems and clinical screening instruments.

## 1. Introduction

Human balance is a complex ability to achieve postural stability, which counteracts the inherently unstable perturbations and body sways induced by the gravitational effect [1]. An efficient balance control depends on the visual, vestibular, somatosensory, muscular, and nervous systems. Assessing human balance helps evaluate the integrity of these systems. In this regard, it is well known that the aging process involves a reduction in physiological capacities and balance [2]. These conditions usually lead to falls which directly and negatively impact older adults’ quality of life [3]. According to the World Health Organization (WHO), 28–35% of the elderly population (above 65 years old) fall each year, reaching 32–42% for adults over 70 years old. This means that the frequency of falls increases with age and frailty level [4]. The relevance of this public health problem is remarkable due to the accelerated growth of the world population of older adults, the intrinsic and extrinsic multifactorial nature of falls [5], and the negative economic impact of attending to the problem, both personally and for governmental health institutions and systems [6,7]. Thus, the correct and timely diagnosis regarding balance anomalies can lead to clinical actions to avoid their impact.

Current balance assessment is based on clinical screening tools and technological systems [8]. The former comprise functional tests of gait, strength, balance, posture, and physical examination that allow the evaluator to diagnose the postural condition and predict patients’ fall risk. However, performance and predictive validity have been questionable because these instruments lack sufficient precision [9]. On the other hand, these tools are not equivalent. They must be selected according to the evaluator’s experience and the clinical context, so their application is usually subjective [10]. These limitations can be reduced using technologies such as instrumented insoles, camera systems, and force platforms. Currently, force platforms are considered the gold standard in clinical practice. These instruments can register stability limits and measure the trajectory of the center of pressure (CoP) [11]. The latter is a biomechanical point where the ground reaction forces are located and concentrated when a person is standing on a flat surface. The CoP measurement allows the characterization of the body sway using metrics and graphs. Despite the usefulness of these tools, they are not routinely used due to their relatively high cost, the complexity of their use, and the reduced portability. Usually, only specialized gait and balance clinics can afford these technologies.

In recent years, the Wii Balance Board (WBB), a device designed for video games, has drawn the attention of scientists and health professionals involved in functional assessment and physical rehabilitation due to its accessibility, cost, portability, and duration of the evaluation [12]. Since its release in 2007, interest in using the WBB for research purposes has grown, the word “Wii-search” was coined [13], and custom-designed modifications, applications, and software were made to use alongside the device [14,15,16,17,18]. It has been used for balance assessment [19] in the fields of neurorehabilitation [13], balance training, and balance and fall risk assessment applications [13,20,21]. It has proven to be an affordable alternative to laboratory-grade force platforms [18,20,22,23], valid and reliable for assessing standing balance [18,19], and precise and reliable for body stability quantitative measures in healthy older women [24].

However, some studies have reported limitations regarding the use of the WBB due to the unstable sampling rate (time jitter), data transmission, low signal-to-noise ratio, and occasional missing data in the acquisition process (glitches in the data) [23]. Additionally, concerns have been raised regarding the accuracy of the WBB sensors, the applicability and practicalities of using the WBB in a clinical setting, and the quality control in the manufacturing process [25,26,27,28,29,30,31,32].

In 2020, the design and construction of a portable electronic device based on a modified WBB (mWBB) capable of measuring and evaluating the balance of human equilibrium were reported [20]. The mWBB is an integrated-embedded device that does not require additional peripherals, such as computers or interfaces, to perform a balance assessment. After modifying the internal electronics of the WBB, the mWBB resolves the technical problems mentioned above: time jitter, data transmission, low signal-to-noise-ratio, and glitches in the data. The technical specifications of the WBB modifications (sensors, signal conditioning, processing, user interface, and data storage) can be found in [20]. These modifications were conducted for research purposes and are based on reverse engineering for interoperability interfacing by developing an electronic module. The practice of reverse engineering is legal, does not violate any patent copyrights, and does not require permission from Nintendo [33,34].

Our previous research verified the criterion validity of the mWBB and its capability to quantify balance deficits in older adults [35]. However, evaluating its intra-rater and inter-rater reliability is necessary when assessing static standing balance. Additionally, it is important to assess if health professionals would find it useful for their general practice. Thus, the present study aims to evaluate the reliability of the CoP indices obtained by the mWBB and to explore the device’s usability.

## 2. Materials and Methods

### 2.1. Study Population

Participants were recruited voluntarily from different nursing homes, universities, and neighborhoods of the cities of Toluca, Metepec, and Villa Guerrero in the State of Mexico, Mexico. Persons eligible to participate were those aged 65 years and over, who could stand for at least 2 min, even using assistive devices. Individuals who drank alcoholic beverages or coffee in the last 24 h or could not complete the physical performance tests (described below) were excluded. The mWBB raters were invited through an open call at the School of Medicine and the School of Nursing and Obstetrics of the Autonomous University of the State of Mexico. All raters were undergraduate students undertaking a bachelor’s degree or had an upper degree in gerontology, physical therapy, nursing, or geriatrics, and had over one year experience in geriatric care and management.

### 2.2. Variables

A total of 78 CoP indices (39 with open-eyes and 39 with closed-eyes) previously described [36] were estimated using the mWBB. Table A1 contains the description of the CoP indices used in this study. For this purpose, subjects were placed on the platform surface with their feet together (closely positioned, side by side, and no opening angle), barefoot, assuming the most upright posture possible, with the arms crossed over the chest [37]. Individuals were asked to focus on a fixed point in front, located half a meter apart in the distance and at a height of 1.5 m above the ground. Participants stood on the mWBB; after a 5-s countdown, the device automatically records the CoP data for one minute. Immediately after, through an auditory stimulus, the subjects were instructed to close their eyes, recording another minute. The test was carried out once. The CoP trajectory data were recorded at a stable sampling rate of 50 Hz, with a resolution of 1/100th of a millimeter and saved in a MicroSD card.

Age in years was used as a continuous variable and sex as a dichotomic variable (woman/man) to describe the sample. Anthropometry (height in cm and weight in kg) was determined following validated methodology and by standardized personnel.

Gait was assessed by the time in seconds taken to complete the Timed Up and Go (TUG) test [38]. Gait deficit (yes/no) was defined when participants took 12 s or more to complete the test. Leg strength was assessed by the number of full stands achieved when performing the 30-s Chair Stand test, and strength in legs deficit (yes/no) was adjusted by sex and age [39]. Balance was assessed with the 4-Stage Balance Test; a balance deficit (yes/no) was present if the individual could not hold their feet-together, semi-tandem, and in tandem positions for ten seconds without moving the feet or needing support, or when participants could not maintain the one-legged stance for five seconds [40].

The use of gait assistive devices, the presence of lower limb prostheses, complete or partial visual and hearing impairments, diagnosis of diabetes or hypertension, fear of falling (FES-I score ≥ 23 [41]) and if the participants fell in the previous year of the study (yes/no) were also analyzed.

The usability of the mWBB was assessed with a custom System Usability Scale (SUS) questionnaire (see Table A2). It has a continuous scale ranging from 0 to 100, administered to all raters immediately upon completion of the reliability tests. The age of the raters, years of experience in geriatric care and management, profile, and score of the SUS test were also recorded.

### 2.3. Reliability

All raters gave standardized instructions to the participants on each trial for the reliability tests. Intra-rater reliability (also known as test–retest reliability) consisted of the same examiner applying the balance test to the same participants twice but at different days in the same room. Based on a previous systematic review [19], the time between the test and retest used for the present study was the closest to 48 h.

Several examiners applied the balance test to the same participants for inter-rater reliability. Each rater repeated one test within an interval closest to 48 h in the same room and the order of raters was randomized [19].

### 2.4. Statistical Analysis

A descriptive analysis of the sample characteristics, the 78 CoP indices for the reliability tests, the characteristics of the raters, and the results of the usability questionnaire was performed. Continuous variables were represented using means and standard deviations (SD), and categorical variables were expressed as numbers and percentages. The normality of the continuous variables was assessed using a Shapiro–Wilk test with α = 0.05. Comparisons of individuals included in the intra-rater and inter-rater tests were estimated through a Wilcoxon test for continuous variables, and a χ^2^ test for categorical variables.

For the intra-rater reliability tests, comparisons of the 78 CoP indices of the test vs. the retest were performed using a t-test for dependent variables for indices with normal distribution. A Wilcoxon test was used for non-parametric indices. To measure the test–retest reliability of the normally distributed CoP indices, Pearson’s correlation coefficient (PCC), and intraclass correlation coefficient (ICC) at 95% confident intervals based on a single rater/measurement, absolute agreement, and two-way mixed effects model [42], were estimated. For those CoP indices that are not normally distributed, Spearman’s Correlation Coefficient (SCC) and ICC at 95% confidence intervals were estimated by the bootstrap technique.

For the inter-rater reliability, a Maulchy’s W test was used to check sphericity. Comparisons of the 78 CoP indices among raters were performed using a Friedman test for non-parametric indices. For normally distributed indices with homogeneity of variances, a dependent variables one-way ANOVA test was used by the Pillai trace statistic. For metrics with normal distribution and heterogeneity of variances, a dependent variables one-way ANOVA test was performed by the Greenhouse–Geisser statistic. To measure the test reliability of the normally distributed CoP indices, PCC, intraclass correlation coefficient (ICC (2,1)) and their 95% confident intervals based on a single measurement, absolute agreement, and two-way random effects model [29] were estimated. For those CoP indices that were not normally distributed, the SCC, ICC (2,1), and their 95% confidence intervals were calculated by the bootstrap technique.

For the usability tests, the correlation between age and years of experience in geriatric care and management versus SUS scores was calculated by the SCC. For the estimation of the degree of usability, SUS scores between 50 and 70 indicate deficient usability, SUS scores above 70 indicate acceptable usability, and values above 90 indicate excellent usability [43,44].

For the reliability tests, it was assumed that 95% confidence interval limits of the ICC below 0.5 indicate poor reliability, values between 0.5 and 0.75 indicate moderate reliability, values between 0.75 and 0.9 indicate good reliability, and values above 0.90 indicate excellent reliability [42].

For Spearman and Pearson correlation coefficients, it was assumed that values between 0.90 and 1.00 indicate very high correlation, values between 0.70 and 0.90 high correlation, values between 0.50 and 0.70 moderate correlation, values between 0.30 and 0.50 low correlation, and values between 0.00 and 0.30 indicate insignificant correlation [45].

The discrimination accuracy of presenting a balance deficit for the 78 CoP indices was assessed using the Hosmer–Lemeshow Goodness of Fit test and the area under the receiver-operating characteristic curve (AUC). The optimal cut-off points were obtained for the indices with the higher AUC that best distinguished between people with and without a balance deficit based on Youden’s statistic. The accuracy of the classification was evaluated with the AUC, sensitivity, and specificity.

All statistical tests were performed with α = 0.05 using IBM SPSS Statistics (version 26.0, Armonk, NY, USA), except for the bootstrap technique and the discrimination accuracy run in Stata Statistical Software (version 15, College Station, TX, USA).

### 2.5. Sample Size Calculation

The sample size for the test–retest reliability was calculated using the correlation coefficient formula (Equation (1)) [46]:

(1) $$n_{TRT}={\left(\frac{z_{\alpha }+z_{\beta }}{0.5\;\ln\frac{1+r}{1-r}}\right)}^{2}+3.$$

where:

$n_{TRT}$ is the sample size for the test–retest reliability,
$z_{\alpha }=1.64$, assuming a 95% confidence level,
$z_{\beta }=1.44$, assuming a *β* error of 0.075, and
r=0.70, is the expected correlation coefficient.

This calculation resulted in 16 participants needed to achieve the desired correlation coefficient.

For the estimation of inter-rater reliability, it is necessary to establish the *rho* (ρ) level, the proportion of variation between subjects in relation to the total variation [47]. The sample size can be calculated by using Equation (2):

(2) $$n_{IR}=\frac{8z_{\alpha /2}^{2}{\left(1-\bar{\rho }\;\right)}^{2}{\left(1+\left(n-1\right)\bar{\rho }\right)}^{2}}{\omega^{2}n\left(n-1\right)}+1.$$

where:

$n_{IR}$ is the sample size for the inter-rater reliability,
$z_{\alpha /2}=1.96$, assuming a 95% confidence level,
$\bar{\rho }=0.70$, is the expected correlation coefficient,
ω=0.25, is the width of the confidence interval,
n=3, is the number of examiners.

This formula resulted in 43 participants needed to achieve the desired correlation coefficient.

## 3. Results

In total, 19 individuals aged 65 and older took part in the intra-rater reliability tests. One participant dropped out of the study, and two were excluded because they could not complete the physical performance tests. Therefore, 16 individuals were included in the test–retest reliability analysis. The mean age of these participants was 75.7 (SD 7.6) years and 56.3% of the sample were women. In total, 13 (81.3%) of all individuals presented gait and balance deficits, and 4 (25%) used assistive gait devices and lower limb prostheses. A total of 3 participants (18.3%) reported visual and hearing impairments. The complete sample had a leg strength deficit. Diabetes was present in 37.5%, fear of falling in 56.3%, and 56.3% of the individuals suffered a fall in the previous year.

Of the 46 individuals who participated in the inter-rater reliability tests, 2 dropped out of the study, and 1 did not complete the physical performance tests. Thus, 43 individuals, of whom 19 (44.2%) were women, were included in the inter-rater analysis. The mean age was 72.6 (SD 7.9) years. In total, 27 individuals (62.8%) presented a gait deficit, 30 (69.8%) a balance deficit, 27 (62.8%) had a fear of falling, 21 (48.8%) reported having suffered a fall in the previous year, and 12 (27.9%) were diagnosed with hypertension. All participants had a leg-strength deficit. No significant difference was found between people who participated in the intra-rater tests and individuals who participated in the inter-rater tests. A complete description of the samples is shown in Table 1.

For intra-rater reliability, there was no significant difference in any CoP index between the test and retest mean values (see Table A3). Table 2 shows the 17 indices with ICC higher than 0.80. The CoP indices with the best level of reliability in the intra-rater tests are POWER95MLCE (ICC = 0.948 and PCC = 0.966), MVELMLOE (ICC = 0.920 and PCC = 0.926), and RDISTMLOE (ICC = 0.883 and PCC = 0.880). A total of 41 indices (52.6%) presented an ICC higher than 0.7 and a correlation coefficient higher than 0.7 (see Table A4 for full results).

For the inter-rater reliability, there was no significant difference in all COP indices between the three examiners, except for MFREQOE, POWER50APOE, FREQDMLOE and FREQDAPOE (see Table A5 for complete results). Table 3 shows the 11 indices with ICC (2,1) higher than 0.75. The CoP indices with the best level reliability in the inter-rater tests are CFREQAPCE (ICC(2,1) = 0.825 (0.717–0.934)), MFREQAPCE (ICC(2,1) = 0.819 (0.711–0.927)) and POWER95APCE (ICC(2,1) = 0.809 (0.701–0.918)). When comparing the three examiners, 30 indices (38.46%) presented an ICC (2,1) higher than 0.7. The correlation coefficient was higher than 0.7 for 46 indices (59.0%) when comparing Rater 1 vs. Rater 2, for 25 indices (32.05%), when comparing Rater 1 vs. Rater 3, and for 23 indices (29.5%) and when comparing Rater 2 vs. Rater 3 (the complete set of the reliability results can be consulted in Table A6).

Three gerontology students and two physiotherapists participated in the usability study. The rater that performed the test–retest trials also attended as one of the three evaluators in the inter-rater test (Rater 1 in Table 3 and Table 4). The two evaluators (Raters 4 and 5) who participated in our previous study [35] also responded to the SUS questionnaire. The five female raters (age: 25.8 (SD 7.12) years; experience in geriatric care and management: 5.1 (SD 3.69) years) answered the SUS questionnaire at the end of all the experimental balance tests.

The results indicate that the mWBB has a mean SUS score of 92.5 points and a standard deviation of 6.84 points. On the other hand, 4 out of 5 raters rank the usability of the WBB as excellent. Only one operator indicated that the WBB has acceptable usability (see the scores in Table 4). The evaluators’ age and years of experience seem not to be related to the SUS scores.

To estimate the discrimination accuracy of the mWBB when presenting a balance alteration, we considered all measurements taken by Rater 1. The first trial of the 16 participants of the intra-rater tests and the results obtained from evaluating the 43 older adults included in the inter-rater tests. A Youden index analysis was run to calculate the optimal cut-off values that provide the best trade-off between sensitivity and specificity for identifying a balance deficit. Then, a ROC analysis was carried out and 10 CoP indices with the highest AUC were obtained (Table 5). The highest AUC was found for the mean frequency of the anterior-posterior CoP time series with eyes open, MFREQAPOE (AUC = 0.778, sensitivity = 0.93, specificity = 0.625). The mean CoP velocity in the anterior-posterior direction and the range of the anterior-posterior CoP presented AUC, sensitivity, and specificity higher than 0.7.

## 4. Discussion

When assessing static balance in a group of individuals aged 65 years and over with a high prevalence of poor physical performance, the most reliable CoP index in the intra-rater tests was the 95% power frequency in the medial-lateral displacement of the CoP with closed-eyes (POWER95MLCE). It had an excellent reliability with an ICC = 0.948 (0.862–0.982) and a PCC = 0.966. The best index for the inter-rater reliability was the centroidal frequency in the anterior-posterior direction with closed-eyes (CFREQAPCE), which had an ICC (2,1) = 0.825. The mWBB also obtained an excellent average usability score of 92.5, showing that the examiners found it useful and easy to use. They will recommend it to other health professionals, regardless of their age or professional experience.

The key indicators when measuring an instrument’s quality are validity and reliability [48]. The first estimates the extent to which a measure agrees with the gold standard. Thus, it has been demonstrated that the WBB is a valid instrument that performs comparably to a laboratory-grade force platform for static standing computerized posturography [18]. Furthermore, previous research showed that the mWBB is a valid device that identifies balance alterations in independent, active older adults with no acute condition. Seventy-three percent of the CoP indices obtained with the mWBB were able to detect balance alterations, with the mean velocity of the CoP in the antero-posterior direction with open-eyes (MVELAPOE) being the best at discriminating between groups [35].

Reliability is defined by the consistency among successive measurements of a variable, on the same subject, and under similar conditions [49]. Some of the instrument’s most critical reliability tests are inter-device, intra-rater, and inter-rater reliability.

Inter-device reliability refers to the consistency of measurements carried out by different devices. Several studies have shown that the WBB presents low inter-device variability [23]. Even after years of use, these devices do not present significant alterations in their measurements, and the battery charge level does not affect the sensor data [50].

Intra-rater reliability refers to the consistency of measurements performed under similar assessment conditions at two separate times by the same examiner (test–retest). On the other hand, inter-rater reliability points to the consistency of measurements carried out by different examiners. Previous evidence [18] has indicated that the WBB is a reliable, safe, and feasible tool to assess static balance in highly functional individuals [51], older adults at risk of falls [52], and adults with stroke [53]. The primary reported drawback of using the WBB for medical assessment is the inconsistent sampling frequency [19]. However, in the design of the mWBB, this problem was addressed and solved [20]. The number of available variables derived from the trajectory of the CoP recorded in quiet stand varies greatly in the literature [54,55]. Most studies only analyze the total length of the CoP path and velocity in stance (time-domain “distance” measures), but further analysis of the other CoP indices can be useful to improve the reliability results, as shown in the present study, where time-domain “area”, time-domain “hybrid”, and frequency domain measures appear between the most reliable indices [36].

It is important to note that there was a high prevalence of physical deficit in both reliability test groups. All the participants presented strength deficits, and over 60% of the sample showed gait and balance deficits. The decline in balance with increased gait variability and lower limb strength [56,57] is associated with an increased risk of falls, resulting in measurements varying wildly from test to test. Despite this, the reliability results of the mWBB corroborate the hypothesis that it is a reliable instrument for assessing the balance in older adults.

For the inter-rater reliability, it is interesting to notice that four indices showed significant differences between raters. Comparisons between pairs of raters indicated that the number of highly correlated indices decreased when comparing Raters 1 and 2 with Rater 3 (see Table A6). The repeatability of the tests could be affected by the degree of the physical decline of the participants. Additionally, the little experience of the raters attending older adults with these characteristics also affected these results. Specifically, Rater 3 had shorter experience in geriatric care.

Our results showed that the CoP indices in the ML direction are the most reliable for intra-rater tests (Table 2). On the other hand, the parameters in the AP direction indicated greater reliability for the inter-rater tests (Table 3). The direction of the variation of the CoP indices depends on the muscles involved in maintaining balance and the contribution of the joints to postural oscillations [55,58]. Clinical and anthropometric factors influencing the CoP variables include sex, presence of vestibular impairments, comorbidities, height, weight, maximum foot width, base of support area, and foot opening angle [55,59]. However, as shown in Table 1, no significant difference was found in the characteristics between the individuals in the two samples. Therefore, given the high degree of physical deterioration of the participants, other features affect the sway direction in both reliability tests. Future research should include variables that affect balance in older people, such as the presence of dementia, depression, sarcopenia, or frailty [60,61,62,63].

Usability is one of the crucial requirements for health technology [64]. The System Usability Scale (SUS) is frequently used because of its validity and availability and its easy score interpretation. However, it is important to notice that it is a weak indicator of critical and severe usability issues compared to the task completion rates. It is a subjective evaluation instrument and only provides a general score of the usability [65]. Furthermore, a larger sample size of evaluators is needed to generalize the results. Therefore, despite the high usability score obtained by the mWBB, further research is needed to establish its use among health professionals who care for older adults.

Due to the high variability between methodological variables, there is no universal consensus on which CoP indices are the best to assess balance and risk of falling [35,55]. The majority of studies show AUC values between 0.7 and 0.8, most of them presenting sensitivity or specificity below 0.7 [35,66,67,68,69,70,71] (comparisons between studies can be found in [35]). Therefore, it is interesting to note that for the classification accuracy, the mean CoP velocity in the anterior-posterior direction with open-eyes (MVELAPOE) and the range of the anterior-posterior CoP with open-eyes (RANGEAPOE) presented: AUC = 0.747, sensitivity = 0.744, and specificity = 0.75 (equality of values for both indices is a coincidence). Furthermore, in our previous study of predictive validity [35], MVELAPOE had the best value of AUC to identify a balance deficit (AUC = 0.714, sensibility = 0.478, specificity = 0.930). We attributed the low level of sensibility to the fact that the studied population in [35] was independent, active, and without any acute conditions. Thus, further research is needed to select indices with high sensibility and specificity in intergroup classifications, depending on the origin of the equilibrium alterations.

Despite all benefits the WBB could bring as a measurement tool in clinical settings [24,72,73], there is an ongoing debate concerning its scientific value [12,25,26,27,28,29,30,31,32]. Some studies have raised concerns about the accuracy of the WBB, the interchangeability of the device with other force platforms, and its use in clinical applications. On the other hand, scientists and clinicians have drawn attention to the need for affordable evaluation tools in non-specialized clinics and less developed countries, regular follow-ups to adapt treatment according to the patient’s performance, and access to tools to prevent the risk of falls. The mWBB presented in this work aims to contribute to the development of more agile and better-adapted hardware and methods that can be available to more patients than current high-end solutions by solving the technical drawbacks of the WBB, and by demonstrating its capability to quantify balance deficits in older adults and the reliability of its measurements.

This study has some limitations. First, reliability tests should be performed under similar assessment conditions and the high degree of physical deterioration of the participants could have affected the tests. However, the results showed which indices were the most appropriate to assess older adults with these characteristics. Second, the difference between the years of experience of the evaluators could have affected the inter-rater reliability tests. Third, a larger sample of experienced personnel is required to generalize the usability results. Fourth, a larger sample is needed to verify the classification accuracy. Finally, like most mass-produced technology, the WBB has a defined life cycle of availability and Nintendo is no longer producing it. However, as prior research has shown similar results between new and used WBBs [74], old platforms could still be used for physical function assessments. Furthermore, the same principle used on these boards is used in electronic bath scales still widely used and produced; these devices are also susceptible to be modified to serve as low-cost balance assessment devices.

## 5. Conclusions

Adding to the literature on the WBB as an acceptable, low-cost, portable, easy to use, and valid device for balance measurement, the mWBB is a reliable device to quantify the CoP displacement during balance tests in older adults, capable of discriminating between people with and without balance deficits.

## Figures and Tables

**Table 1:** Characteristics of older adults that participated in the reliability tests.

Characteristic	Intra-Rater *n* = 16	Inter-Rater *n* = 43	*p*-Value
Age (years)	75.66 (7.62)	72.61 (7.86)	0.264
Sex (women)	9 (56.3%)	19 (44.2%)	0.409
Height (cm)	154.68 (9.89)	158.83 (11.17)	0.191
Weight (kg)	59.79 (9.54)	65.09 (10.82)	0.100
Gait deficit	13 (81.3%)	27 (62.8%)	0.177
Leg-strength deficit	16 (100%)	43 (100%)	-
Balance deficit	13 (81.3%)	30 (69.8%)	0.378
Use of gait assistive devices	4 (25%)	9 (20.9%)	0.737
Presence of lower limb prostheses	4 (25%)	6 (14%)	0.315
Complete or partial visual impairment	3 (18.3%)	4 (9.3%)	0.318
Partial hearing impairment	3 (18.3%)	3 (7%)	0.183
Diabetes	6 (37.5%)	10 (23.3%)	0.274
Hypertension	4 (25%)	12 (27.9%)	0.823
Fear of falling (FES-I score ≥ 23)	9 (56.3%)	27 (62.8%)	0.647
Fell last year	9 (56.3%)	21 (48.8%)	0.613

Continuous variables are presented as Means and Standard Deviation (SD); categorical variables are presented as Frequencies (percentages). For intra-rater variables, there were one missing data for age and weight. For inter-rater variables, there were one missing data for age and height and two missing data for weight.

**Table 2:** Statistical analysis of CoP indices with the best level of reliability in intra-rater (test–retest) reliability.

CoP Indices	Test Mean (SD)	Retest Mean (SD)	ICC (IC 95%)	Correlation Coefficient
POWER95MLCE	1.5 (0.82)	1.59 (0.99)	0.948 (0.862–0.982)	0.966
MVELMLOE	9.65 (7.25)	10.49 (8.04)	0.920 (0.792–0.971)	0.926
RDISTMLOE	4.41 (2.4)	4.98 (2.49)	0.883 (0.640–0.951)	0.880
RDISTOE	6.72 (3.37)	7.22 (3.08)	0.882 (0.572–0.993)	0.826
POWER95RDOE	2.08 (0.65)	2.09 (0.81)	0.869 (0.665–0.952)	0.884
sRDOE	3.39 (1.81)	3.48 (1.51)	0.868 (0.664–0.952)	0.879
TPOWERMLOE	12.43 (16.54)	12.39 (17.96)	0.859 (0.640–0.949)	0.854
RDISTAPCE	6.08 (2.58)	5.9 (2.3)	0.852 (0.629–0.945)	0.851
MDISTAPCE	4.85 (2.03)	4.69 (1.83)	0.851 (0.629–0.945)	0.851
RANGEMLOE	25.36 (15.46)	28.23 (17.17)	0.834 (0.597–0.938)	0.843
CFREQMLOE	0.72 (0.24)	0.7 (0.21)	0.834 (0.594–0.939)	0.836
MDISTMLOE	3.45 (1.83)	3.96 (1.98)	0.832 (0.563–0.939)	0.857
CFREQMLCE	0.74 (0.25)	0.79 (0.36)	0.819 (0.565–0.932)	0.873
POWER95MLOE	1.44 (0.82)	1.37 (0.68)	0.817 (0.638–0.996)	0.604
AREACCOE	507.37 (497.54)	534.65 (497.19)	0.817 (0.637–0.996)	0.774
AREACEOE	480.76 (467.52)	492.03 (501.33)	0.815 (0.545–0.932)	0.807
MVELAPCE	21.84 (14.39)	22.66 (11.35)	0.809 (0.622–0.996)	0.776

All *p*-values of the correlation coefficients ≤ 0.001. For the intra-rater reliability, there was not a significant difference in any CoP indices between test and retest mean values. See Appendix C for the full results.

**Table 3:** Statistical analysis of CoP indices with the best level of reliability in the inter-rater.

CoP Indices	Rater 1 Mean (SD)	Rater 2 Mean (SD)	Rater 3 Mean (SD)	ICC(2,1) (CI 95%)
CFREQAPCE	0.84 (0.3)	0.87 (0.28)	0.84 (0.26)	0.825 (0.717–0.934)
MFREQAPCE	0.69 (0.36)	0.68 (0.28)	0.69 (0.33)	0.819 (0.711–0.927)
POWER95APCE	1.71 (0.76)	1.74 (0.65)	1.71 (0.69)	0.809 (0.701–0.918)
MVELAPOE	12.37 (7.77)	11.44 (7.07)	10.93 (5.84)	0.789 (0.665–0.914)
MVELOE	17.02 (8.98)	15.88 (8.48)	15.74 (8.08)	0.789 (0.676–0.901)
POWER95RDCE	1.98 (0.69)	2.04 (0.6)	2.06 (0.69)	0.774 (0.660–0.861)
AREASWOE	32.94 (27.16)	30.43 (30.14)	31.51 (33.39)	0.768 (0.604–0.932)
FDCCCE	1.93 (0.2)	1.92 (0.15)	1.93 (0.18)	0.766 (0.648–0.856)
POWER50APCE	0.44 (0.15)	0.46 (0.14)	0.45 (0.15)	0.762 (0.622–0.902)
MVELAPCE	17.64 (10.32)	16.3 (8.69)	17.32 (10.98)	0.752 (0.594–0.911)
FDPDCE	1.78 (0.15)	1.76 (0.13)	1.76 (0.13)	0.751 (0.628–0.846)

See Appendix D for the full results.

**Table 4:** Characteristics of raters that participated in the usability study.

ID	Age [Years]	Experience in Geriatric Care and Management [Years]	Professional Profile	SUS Score
Rater 1	21	3.5	Gerontology student	100
Rater 2	21	2.5	Gerontology student	90
Rater 3	20	1.5	Gerontology student	82.5
Rater 4	35	10	Physiotherapist	92.5
Rater 5	32	8	Physiotherapist	97.5

**Table 5:** Statistical analysis of the 10 CoP indices with the highest area under the curve (AUC) related to presenting a balance deficit, optimal cut-off values for the indices, sensitivity and specificity.

CoP Indices	Without Balance Deficit *n* = 16 Mean (SD)	With Balance Deficit *n* = 43 Mean (SD)	Optimal Cut-Off Point	AUC	Sensitivity	Specificity
MFREQAPOE	0.59 (0.52)	0.70 (0.30)	0.4383	0.778	0.93	0.625
AREASWOE	18.50 (15.0)	43.91 (39.24)	15.4771	0.774	0.861	0.688
MVELOE	12.51 (6.92)	20.37 (11.15)	15.8459	0.752	1	0
MVELAPOE	8.64 (5.96)	15.52 (9.34)	10.2551	0.747	0.744	0.75
RANGEAPOE	18.89 (7.53)	28.87 (12.52)	20.3611	0.747	0.744	0.75
RANGEOE	22.54 (10.35)	34.25 (17.18)	22.0363	0.743	0.861	0.625
RANGEXOE	18.36 (9.44)	27.99 (16.29)	18.2051	0.739	0.791	0.688
TPOWERAPOE	6.16 (6.84)	14.93 (19.72)	6.9855	0.732	1	0
sRDOE	2.37 (1.05)	3.43 (1.55)	2.1303	0.723	0.884	0.563
AREACCOE	256.28 (233.49)	486.57 (393.27)	150.5037	0.692	0.884	0.5

## Data Availability

Data bases are anonymized and available as Supplementary Material named database.csv.

## Supplementary Materials

The following supporting information can be downloaded at: https://www.mdpi.com/article/10.3390/ijerph191711026/s1 CSV File: database.csv—Database used for the analysis.

## Appendix A

**Table A1:** Description of the center of pressure (CoP) indices.

CoP Indices	Units	Description	Formula
RD	mm	Resultant Distance time series	$RD\left[n\right]\;=\;{\left[AP{\left[n\right]}^{2}+ML{\left[n\right]}^{2}\right]}^{1/2}\;n\;=\;1,\ldots ,\;N.$
MDIST	mm	Mean Distance	$MDIST=1/N\sum RD\left[n\right]$
MDISTML	mm	Mean Distance in Medial-Lateral displacement	$MDISTML=1/N\sum ML\left[n\right]$
MDISTAP	mm	Mean Distance in Anterior-Posterior displacement	$MDISTAP=1/N\sum AP\left[n\right]$
RDIST	mm	RMS distance value from the mean CoP	$RDIST={\left[1/N\sum RD{\left[n\right]}^{2}\right]}^{1/2}$
RDISTML	mm	RMS distance of the Medial-Lateral time series	$RDISTML={\left[1/N\sum ML{\left[n\right]}^{2}\right]}^{1/2}$
RDISTAP	mm	RMS distance of the Anterior-Posterior time series	$RDISTAP={\left[1/N\sum AP{\left[n\right]}^{2}\right]}^{1/2}$
RANGE	mm	Maximum distance between any two points (*p*_1_, *p*_2_) on the CoP path	$RANGE=Max\left(d\left(p_{1},p_{2}\right)\right)$
RANGEML	mm	Range of Medial-Lateral CoP time series	$RANGEX=Max\left(ML\right)$
RANGEAP	mm	Range of Anterior-Posterior CoP time series	$RANGEY=Max\left(AP\right)$
MVEL	mm/s	Mean Velocity of the CoP	MVELO=TOTEX/T
MVELML	mm/s	Mean CoP Velocity in Medial-Lateral direction	MVELOML=TOTEXML/T
MVELAP	mm/s	Mean CoP Velocity in Anterior-Posterior direction	MVELOAP=TOTEXAP/T
sRD	mm	Standar Deviation of the RD time series	$sRD={\left[RDIST^{2}-MDIST^{2}\right]}^{1/2}$
sAPML	mm^2^	Covariance of Medial-Lateral & Anterior-Posterior data	$sAPML=1/N\sum ML\left[n\right]AP\left[n\right]$
AREACC	mm^2^	95% Confidence Circle Area	$AREACC=\pi {\left[MDIST+1.645\;sRD\right]}^{2}$
AREACE	mm^2^	95% Confidence Ellipse Area	$AREACE=6\pi {\left[RDISTAP^{2}\;RDISTML^{2}-sAPML^{2}\right]}^{1/2}$
AREASW	mm^2^/s	Sway Area of the CoP path per unit of time	$AREASW=1/2T\sum_{n=1}^{N-1}\left[AP\left[n+1\right]ML\left[n\right]-ML\left[n+1\right]AP\left[n\right]\right]$
MFREQ	Hz	Mean Frequency of CoP	$MFREQ=MVELO/\left(2\pi MDIST\right)$
MFREQML	Hz	Mean Frequency of Medial-Lateral CoP time series	$MFREQML=MVELOML/\left(4\sqrt{2}MDISTML\right)$
MFREQAP	Hz	Mean Frequency of Anterior-Posterior CoP time series	$MFREQAP=MVELOAP/\left(4\sqrt{2}MDISTAP\right)$
FDPD	-	Fractal dimension that models the area of the stabilogram with a circle of diameter *d* = range	$FDPD=\log\left(N\right)/\log\left(Nd/TOTEX\right)$
FDCC	-	Fractal dimension based on AREACC	$FDCC=\log\left(N\right)/\log\left(2N\left(MDIST+1.645sRD\right)/TOTEX\right)$
FDCE	-	Fractal dimension based on AREACE	$FDCE=\log\left(N\right)/\log\left(N{\left(24{\left[RDISTAP^{2}\;RDISTML^{2}-SAPML^{2}\right]}^{1/2}\right)}^{1/2}/TOTEX\right)$
TPOWERRD	mm^2^/Hz	Total Power of CoP Resultant Distance data	$\mu_{0}=\sum_{m=3}^{100}G_{RD}\left(m\right)$
TPOWERML	mm^2^/Hz	Total Power of CoP Medial-Lateral data	$\mu_{0ML}=\sum_{m=3}^{100}G_{ML}\left(m\right)$
TPOWERAP	mm^2^/Hz	Total Power of CoP Anterior-Posterior data	$\mu_{0AP}=\sum_{m=3}^{100}G_{AP}\left(m\right)$
POWER50RD	Hz	50% Power Frequency of Resultant Distance CoP data	$POWER50RD=\sum_{m=3}^{u}G_{RD}\left(m\right)\geq 0.5\mu_{0}$
POWER50ML	Hz	50% Power Frequency of Medial-Lateral CoP data	$POWER50ML=\sum_{m=3}^{u}G_{ML}\left(m\right)\geq 0.5\mu_{0ML}$
POWER50AP	Hz	50% Power Frequency of Anterior-Posterior CoP data	$POWER50AP=\sum_{m=3}^{u}G_{AP}\left(m\right)\geq 0.5\mu_{0AP}$
POWER95RD	Hz	95% Power Frequency of Resultant Distance CoP data	$POWER95RD=\sum_{m=3}^{u}G_{RD}\left(m\right)\geq 0.95\mu_{0}$
POWER95ML	Hz	95% Power Frequency of Medial-Lateral CoP data	$POWER95ML=\sum_{m=3}^{u}G_{ML}\left(m\right)\geq 0.95\mu_{0ML}$
POWER95AP	Hz	95% Power Frequency of Anterior-Posterior CoP data	$POWER95AP=\sum_{m=3}^{u}G_{AP}\left(m\right)\geq 0.95\mu_{0AP}$
CFREQRD	Hz	Centroidal Frequency of Resultant Distance CoP data	$CFREQRD={\left(\sum_{m=3}^{100}{\left(m\Delta f\right)}^{2}G_{RD}\left(m\right)/\;\mu_{0}\right)}^{1/2}\;$
CFREQML	Hz	Centroidal Frequency of Medial-Lateral CoP data	$CFREQML={\left(\sum_{m=3}^{100}{\left(m\Delta f\right)}^{2}G_{ML}\left(m\right)/\mu_{0ML}\;\right)}^{1/2}$
CFREQAP	Hz	Centroidal Frequency of Anterior-Posterior CoP data	$CFREQAP={\left(\sum_{m=3}^{100}{\left(m\Delta f\right)}^{2}G_{AP}\left(m\right)\;/\;\mu_{0AP}\;\right)}^{1/2}$
FREQDRD	-	Frequency Dispersion of Resultant Distance CoP data	$FREQDRD={\left(\left(1-{\left(\sum_{m=3}^{100}\left(m\Delta f\right)G_{RD}\left(m\right)\right)}^{2}\right)/\mu_{0}\left(\sum_{m=3}^{100}{\left(m\Delta f\right)}^{2}G_{RD}\left(m\right)\right)\right)}^{1/2}\;$
FREQDML	-	Frequency Dispersion of Medial-Lateral CoP data	$FREQDML={\left(\left(1-{\left(\sum_{m=3}^{100}\left(m\Delta f\right)G_{ML}\left(m\right)\right)}^{2}\right)/\mu_{0ML}\left(\sum_{m=3}^{100}{\left(m\Delta f\right)}^{2}G_{ML}\left(m\right)\right)\right)}^{1/2}\;$
FREQDAP	-	Frequency Dispersion of Anterior-Posterior CoP data	$FREQDAP={\left(\left(1-{\left(\sum_{m=3}^{100}\left(m\Delta f\right)G_{AP}\left(m\right)\right)}^{2}\right)/\mu_{0AP}\left(\sum_{m=3}^{100}{\left(m\Delta f\right)}^{2}G_{AP}\left(m\right)\right)\right)}^{1/2}\;$

*N* is the number of data points included in the CoP time series (*N* = 2400 with open eyes and with closed eyes). *T* is the period of the time selected for analysis (*T* = 48 s in this work). *G*_x *(m)* is the discrete power spectral density (*x* stands for RD, ML or AP). u is the smallest integer that converges in recursive sums.

## Appendix B

**Table A2:** Rater questionnaire on the usability of the modified Wii Balance Board (mWBB).

	Strongly Disagree			Strongly Agree	
1. I think I would like to use the mWBB frequently.	1 ☐	2 ☐	3 ☐	4 ☐	5 ☐
2. I find the mWBB very complex.	1 ☐	2 ☐	3 ☐	4 ☐	5 ☐
3. I think the mWBB is easy to use.	1 ☐	2 ☐	3 ☐	4 ☐	5 ☐
4. I think I would need technical support to make use of the mWBB.	1 ☐	2 ☐	3 ☐	4 ☐	5 ☐
5. I find the various functions of the mWBB well integrated.	1 ☐	2 ☐	3 ☐	4 ☐	5 ☐
6. I think there is too much inconsistency in the mWBB.	1 ☐	2 ☐	3 ☐	4 ☐	5 ☐
7. I think most people would learn to use the mWBB quickly.	1 ☐	2 ☐	3 ☐	4 ☐	5 ☐
8. I find the mWBB quite uncomfortable to use.	1 ☐	2 ☐	3 ☐	4 ☐	5 ☐
9. I feel very confident in using the mWBB.	1 ☐	2 ☐	3 ☐	4 ☐	5 ☐
10. I needed to learn a lot of things before I could operate the mWBB properly.	1 ☐	2 ☐	3 ☐	4 ☐	5 ☐

## Appendix C

**Table A3:** Statistical analysis 78 CoP indices intra-rater.

CoP Indices	Test Mean (SD)	Retest Mean (SD)	*p*-Value Shapiro–Wilk Test	*p*-Value Means Difference Test
RDOE	13,913.74 (6860.32)	15,163.36 (6547.64)	0.810	0.268
MDISTOE	5.79 (2.85)	6.31 (2.72)	0.810	0.268
MDISTMLOE	3.45 (1.83)	3.96 (1.98)	0.638	0.063
MDISTAPOE	3.83 (2.06)	4.09 (1.86)	0.632	0.550
RDISTOE	6.72 (3.37)	7.22 (3.08)	0.629	0.311
RDISTMLOE	4.41 (2.4)	4.98 (2.49)	0.974	0.079
RDISTAPOE	4.91 (2.69)	5.06 (2.27)	0.558	0.772
RANGEOE	33.09 (17.77)	33.54 (15.78)	0.754	0.871
RANGEMLOE	25.36 (15.46)	28.23 (17.17)	0.673	0.237
RANGEAPOE	30.14 (17.27)	27.16 (12.31)	0.967	0.280
MVELOE	21.52 (14.2)	21.97 (13.75)	0.021 *	0.642
MVELMLOE	9.65 (7.25)	10.49 (8.04)	1.000	0.288
MVELAPOE	17.09 (11.39)	16.86 (10.41)	0.002 *	0.918
sRDOE	3.39 (1.81)	3.48 (1.51)	0.056	0.684
sAPMLOE	−3.14 (5.91)	−0.48 (14.53)	0.376	0.524
AREACCOE	507.37 (497.54)	534.65 (497.19)	0.028 *	0.535
AREACEOE	480.76 (467.52)	492.03 (501.33)	0.100	0.883
AREASWOE	47.95 (52.87)	51.52 (68.26)	0.013 *	0.501
MFREQOE	0.58 (0.19)	0.58 (0.35)	0.000 *	0.278
MFREQMLOE	0.49 (0.17)	0.48 (0.2)	0.397	0.817
MFREQAPOE	0.78 (0.33)	0.77 (0.47)	0.030 *	0.438
FDPDOE	1.78 (0.14)	1.78 (0.18)	0.529	0.930
FDCCOE	1.94 (0.17)	1.93 (0.26)	0.062	0.827
FDCEOE	1.95 (0.16)	1.96 (0.25)	0.003 *	0.569
TPOWERRDOE	9.65 (12.65)	8.67 (10.11)	0.000 *	1.000
TPOWERMLOE	12.43 (16.54)	12.39 (17.96)	0.821	0.985
TPOWERAPOE	18.56 (30.14)	13.01 (14.87)	0.000 *	0.836
POWER50RDOE	0.52 (0.13)	0.55 (0.28)	0.006 *	0.816
POWER50MLOE	0.41 (0.1)	0.37 (0.08)	0.024 *	0.265
POWER50APOE	0.53 (0.17)	0.58 (0.24)	0.113	0.389
POWER95RDOE	2.08 (0.65)	2.09 (0.81)	0.496	0.853
POWER95MLOE	1.44 (0.82)	1.37 (0.68)	0.002 *	0.820
POWER95APOE	1.99 (0.68)	2.08 (0.86)	0.315	0.466
CFREQRDOE	1.02 (0.24)	1.03 (0.4)	0.044 *	0.756
CFREQMLOE	0.72 (0.24)	0.7 (0.21)	0.463	0.484
CFREQAPOE	0.98 (0.24)	1.03 (0.39)	0.298	0.408
FREQDRDOE	0.67 (0.04)	0.66 (0.04)	0.878	0.582
FREQDMLOE	0.63 (0.07)	0.64 (0.05)	0.179	0.410
FREQDAPOE	0.66 (0.06)	0.64 (0.05)	0.968	0.297
RDCE	16,923.1 (6812.85)	17,838.53 (7641.53)	0.005 *	0.501
MDISTCE	7.05 (2.83)	7.43 (3.18)	0.005 *	0.501
MDISTMLCE	4.02 (2.13)	4.77 (2.41)	0.138	0.166
MDISTAPCE	4.85 (2.03)	4.69 (1.83)	0.210	0.565
RDISTCE	8.14 (3.37)	8.55 (3.69)	0.023 *	0.642
RDISTMLCE	5.12 (2.82)	6.08 (3.11)	0.118	0.156
RDISTAPCE	6.08 (2.58)	5.9 (2.3)	0.202	0.604
RANGECE	40.48 (18.09)	42.18 (20.48)	0.725	0.644
RANGEMLCE	29.8 (17.87)	36.98 (22.15)	0.788	0.159
RANGEAPCE	35.53 (16.66)	34.04 (12.66)	0.243	0.574
MVELCE	27.49 (18.03)	29.91 (14.18)	0.711	0.375
MVELMLCE	12.42 (8.85)	14.65 (8.18)	0.852	0.213
MVELAPCE	21.84 (14.39)	22.66 (11.35)	0.035 *	1.000
sRDCE	4.06 (1.86)	4.21 (1.94)	0.870	0.694
sAPMLCE	−1.93 (10.24)	0.57 (16.89)	0.300	0.449
AREACCCE	694.38 (495.01)	764.61 (562.87)	0.023 *	0.605
AREACECE	634.3 (471.31)	739.93 (544.89)	0.001 *	0.148
AREASWCE	74.12 (73.45)	79.52 (54.79)	0.020 *	0.569
MFREQCE	0.59 (0.22)	0.68 (0.38)	0.000 *	0.569
MFREQMLCE	0.58 (0.3)	0.57 (0.23)	0.134	0.822
MFREQAPCE	0.77 (0.34)	0.89 (0.56)	0.000 *	0.679
FDPDCE	1.78 (0.13)	1.83 (0.20)	0.220	0.268
FDCCCE	1.95 (0.18)	2.01 (0.27)	0.004 *	0.501
FDCECE	1.98 (0.17)	2.02 (0.26)	0.031 *	0.796
TPOWERRDCE	12.27 (10.66)	12.94 (10.68)	0.280	0.780
TPOWERMLCE	19.79 (21.94)	25.63 (26.64)	0.007 *	0.642
TPOWERAPCE	20.89 (14.7)	20.88 (15.75)	0.996	0.996
POWER50RDCE	0.57 (0.12)	0.66 (0.38)	0.010 *	0.495
POWER50MLCE	0.4 (0.1)	0.39 (0.13)	0.293	0.793
POWER50APCE	0.54 (0.17)	0.54 (0.28)	0.008 *	0.470
POWER95RDCE	2.26 (0.64)	2.3 (0.95)	0.854	0.817
POWER95MLCE	1.5 (0.82)	1.59 (0.99)	0.590	0.225
POWER95APCE	2.04 (0.81)	2.09 (0.84)	0.685	0.754
CFREQRDCE	1.08 (0.23)	1.14 (0.48)	0.115	0.487
CFREQMLCE	0.74 (0.25)	0.79 (0.36)	0.071	0.347
CFREQAPCE	0.99 (0.3)	1.04 (0.42)	0.054	0.524
FREQDRDCE	0.66 (0.03)	0.65 (0.05)	0.455	0.287
FREQDMLCE	0.64 (0.07)	0.65 (0.07)	0.698	0.752
FREQDAPCE	0.66 (0.03)	0.66 (0.04)	0.719	0.574

* *p*-value < 0.05.

**Table A4:** Statistical analysis of intra-rater reliability 78 CoP indices.

CoP Indices	Correlation Coefficient	*p*-Value Correlation Coefficient	ICC (IC 95%)
RDOE	0.791	0.000 *	0.787 (0.502–0.919)
MDISTOE	0.791	0.000 *	0.787(0.502–0.919)
MDISTMLOE	0.857	0.000 *	0.832 (0.563–0.939)
MDISTAPOE	0.606	0.013 *	0.612 (0.182–0.845)
RDISTOE	0.826	0.000 *	0.882 (0.572–0.993)
RDISTMLOE	0.880	0.000 *	0.883 (0.640–0.951)
RDISTAPOE	0.680	0.004 *	0.683 (0.292–0.877)
RANGEOE	0.794	0.000 *	0.799 (0.510–0.925)
RANGEMLOE	0.843	0.000 *	0.834 (0.597–0.938)
RANGEAPOE	0.792	0.000 *	0.745 (0.423–0.902)
MVELOE	0.629	0.009 *	0.743 (0.491–0.995)
MVELMLOE	0.926	0.000 *	0.920 (0.792–0.971)
MVELAPOE	0.594	0.015 *	0.628 (0.263–0.993)
sRDOE	0.879	0.000 *	0.868 (0.664–0.952)
sAPMLOE	-0.118	0.662	0.086 (0.000–0.577)
AREACCOE	0.774	0.000 *	0.817 (0.637–0.996)
AREACEOE	0.807	0.000 *	0.815 (0.545–0.932)
AREASWOE	0.779	0.000 *	0.788 (0.581–0.995)
MFREQOE	0.759	0.001 *	0.627 (0.261–0.993)
MFREQMLOE	0.714	0.002 *	0.719 (0.356–0.893)
MFREQAPOE	0.668	0.005 *	0.561(0.131–0.991)
FDPDOE	0.780	0.000 *	0.772 (0.455–0.995)
FDCCOE	0.802	0.000 *	0.746 (0.406–0.904)
FDCEOE	0.765	0.001 *	0.687 (0.380–0.994)
TPOWERRDOE	0.697	0.003 *	0.660 (0.330–0.990)
TPOWERMLOE	0.854	0.000 *	0.859 (0.640–0.949)
TPOWERAPOE	0.697	0.003 *	0.590 (0.187–0.992)
POWER50RDOE	0.545	0.029 *	0.409 (0.000–0.989)
POWER50MLOE	0.415	0.110	0.492 (0.000–0.990)
POWER50APOE	0.451	0.080	0.426 (0.000–0.753)
POWER95RDOE	0.884	0.000 *	0.869 (0.665–0.952)
POWER95MLOE	0.604	0.013 *	0.817 (0.638–0.996)
POWER95APOE	0.805	0.000 *	0.788 (0.489–0.920)
CFREQRDOE	0.759	0.001 *	0.792 (0.588–0.995)
CFREQMLOE	0.836	0.000 *	0.834 (0.594–0.939)
CFREQAPOE	0.825	0.000 *	0.734 (0.400–0.898)
FREQDRDOE	0.293	0.272	0.300 (0.000–0.687)
FREQDMLOE	0.779	0.000 *	0.762 (0.450–0.909)
FREQDAPOE	0.187	0.489	0.183 (0.000–0.608)
RDCE	0.697	0.003 *	0.789 (0.574–0.995)
MDISTCE	0.677	0.003 *	0.784 (0.573–0.996)
MDISTMLCE	0.602	0.014 *	0.581 (0.162–0.828)
MDISTAPCE	0.851	0.000 *	0.851 (0.629–0.945)
RDISTCE	0.641	0.007 *	0.780 (0.566–0.995)
RDISTMLCE	0.629	0.009 *	0.608 (0.201–0.840)
RDISTAPCE	0.851	0.000 *	0.852 (0.629–0.945)
RANGECE	0.729	0.001 *	0.734 (0.387–0.898)
RANGEMLCE	0.549	0.028 *	0.518 (0.078–0.796)
RANGEAPCE	0.784	0.000 *	0.763 (0.445–0.910)
MVELCE	0.809	0.000 *	0.788 (0.503–0.920)
MVELMLCE	0.677	0.004 *	0.665 (0.288–0.867)
MVELAPCE	0.776	0.000 *	0.809 (0.622–0.996)
sRDCE	0.689	0.003 *	0.700 (0.324–0.884)
sAPMLCE	0.645	0.007 *	0.578 (0.136–0.829)
AREACCCE	0.665	0.005 *	0.622 (0.253–0.991)
AREACECE	0.694	0.003 *	0.599 (0.205–0.992)
AREASWCE	0.771	0.000 *	0.665 (0.337–0.993)
MFREQCE	0.656	0.006 *	0.387 (0.000–0.989)
MFREQMLCE	0.803	0.000 *	0.786 (0.485–0.920)
MFREQAPCE	0.515	0.041 *	0.315 (0.000–0.986)
FDPDCE	0.337	0.150	0.336 (0.000–0.701)
FDCCCE	0.579	0.019 *	0.413 (0.000–0.984)
FDCECE	0.771	0.000 *	0.648 (0.303–0.993)
TPOWERRDCE	0.615	0.011 *	0.629 (0.200–0.854)
TPOWERMLCE	0.732	0.001 *	0.441 (0.000–0.990)
TPOWERAPCE	0.753	0.001 *	0.763 (0.438–0.911)
POWER50RDCE	0.283	0.288	0.257 (0.000–0.986)
POWER50MLCE	0.200	0.457	0.204 (0.000–0.633)
POWER50APCE	0.708	0.002 *	0.488 (0.000–0.990)
POWER95RDCE	0.642	0.007 *	0.611 (0.170–0.846)
POWER95MLCE	0.966	0.000 *	0.948 (0.862–0.982)
POWER95APCE	0.788	0.000 *	0.797(0.509–0.924)
CFREQRDCE	0.640	0.008 *	0.511 (0.037–0.797)
CFREQMLCE	0.873	0.000 *	0.819 (0.565–0.932)
CFREQAPCE	0.802	0.000 *	0.763 (0.447–0.910)
FREQDRDCE	0.487	0.056	0.431 (0.000–0.753)
FREQDMLCE	0.294	0.269	0.306 (0.000–0.692)
FREQDAPCE	0.084	0.757	0.083 (0.000–0.552)

* *p*-value < 0.05.

## Appendix D

**Table A5:** Statistical analysis 78 CoP Indices inter-raters.

CoP Indices	Rater 1 Mean (SD)	Rater 2 Mean (SD)	Rater 3 Mean (SD)	*p*-Value Shapiro–Wilk Test	*p*-Value Mauchly Test	*p*-Value Means Difference Test
RDOE	13,058.98 (4666.8)	13,137.28 (4445.82)	13,303.54 (5218.12)	0.001 *	-	0.739
MDISTOE	5.44 (1.94)	5.47 (1.85)	5.54 (2.17)	0.001 *	-	0.739
MDISTMLOE	3.32 (1.42)	3.27 (1.41)	3.26 (1.55)	0.000 *	-	0.850
MDISTAPOE	3.57 (1.28)	3.69 (1.4)	3.74 (1.59)	0.002 *	-	0.911
RDISTOE	6.26 (2.31)	6.24 (2.1)	6.39 (2.51)	0.000 *	-	0.559
RDISTMLOE	4.27 (1.91)	4.06 (1.69)	4.15 (1.98)	0.000 *	-	0.643
RDISTAPOE	4.48 (1.6)	4.59 (1.7)	4.7 (1.96)	0.005 *	-	0.911
RANGEOE	30.32 (16.01)	29.17 (10.93)	30.57 (13.21)	0.021 *	-	0.614
RANGEMLOE	25.38 (15.41)	22.37 (9.53)	24.19 (13)	0.011 *	-	0.559
RANGEAPOE	24.67 (9.47)	24.89 (9.29)	25.84 (10.76)	0.028 *	-	0.911
MVELOE	17.02 (8.98)	15.88 (8.48)	15.74 (8.08)	0.000 *	-	0.368
MVELMLOE	9.02 (3.8)	8.58 (4.09)	8.94 (5.07)	0.000 *	-	0.327
MVELAPOE	12.37 (7.77)	11.44 (7.07)	10.93 (5.84)	0.000 *	-	0.404
sRDOE	3.04 (1.37)	2.98 (1.03)	3.16 (1.3)	0.000 *	-	0.521
sAPMLOE	−0.3 (6.98)	0.56 (9.96)	1.78 (5.86)	0.014 *	-	0.231
AREACCOE	393.14 (311.15)	376.14 (291.78)	418.76 (346.14)	0.000 *	-	0.811
AREACEOE	381.28 (284.2)	351.21 (282.74)	394.51 (330.28)	0.000 *	-	0.739
AREASWOE	32.94 (27.16)	30.43 (30.14)	31.51 (33.39)	0.000 *	-	0.320
MFREQOE	0.51 (0.25)	0.46 (0.18)	0.45 (0.13)	0.827	0.003 *	0.050 *
MFREQMLOE	0.52 (0.21)	0.49 (0.16)	0.5 (0.16)	0.314	0.163	0.532
MFREQAPOE	0.62 (0.37)	0.55 (0.27)	0.53 (0.18)	0.533	0.052	0.106
FDPDOE	1.73 (0.14)	1.71 (0.13)	1.70 (0.11)	0.342	0.711	0.105
FDCCOE	1.88 (0.19)	1.84 (0.16)	1.84 (0.12)	0.166	0.137	0.096
FDCEOE	1.88 (0.19)	1.86 (0.15)	1.85 (0.13)	0.926	0.019 *	0.092
TPOWERRDOE	6.65 (5.67)	5.86 (5.01)	7.42 (7.04)	0.000 *	-	0.534
TPOWERMLOE	10.03 (8.78)	8.62 (10.03)	10.4 (13.03)	0.000 *	-	0.811
TPOWERAPOE	10.31 (9.12)	10.09 (10.49)	12.01 (13.45)	0.000 *	-	0.643
POWER50RDOE	0.51 (0.18)	0.5 (0.15)	0.47 (0.14)	0.209	0.021 *	0.119
POWER50MLOE	0.42 (0.14)	0.41 (0.11)	0.38 (0.1)	0.211	0.061	0.172
POWER50APOE	0.47 (0.17)	0.43 (0.13)	0.4 (0.12)	0.162	0.456	0.017 *
POWER95RDOE	1.81 (0.63)	1.85 (0.64)	1.79 (0.51)	0.471	0.236	0.780
POWER95MLOE	1.29 (0.5)	1.3 (0.48)	1.31 (0.35)	0.178	0.565	0.924
POWER95APOE	1.61 (0.77)	1.59 (0.63)	1.51 (0.54)	0.223	0.655	0.478
CFREQRDOE	0.91 (0.28)	0.92 (0.27)	0.89 (0.22)	0.015 *	-	0.404
CFREQMLOE	0.69 (0.21)	0.69 (0.19)	0.67 (0.15)	0.990	0.105	0.703
CFREQAPOE	0.83 (0.33)	0.79 (0.25)	0.76 (0.21)	0.369	0.117	0.171
FREQDRDOE	0.64 (0.04)	0.64 (0.03)	0.65 (0.04)	0.799	0.464	0.170
FREQDMLOE	0.6 (0.05)	0.61 (0.04)	0.63 (0.05)	0.106	0.513	0.024 *
FREQDAPOE	0.64 (0.05)	0.65 (0.04)	0.66 (0.05)	0.845	0.742	0.040 *
RDCE	17,296.17 (6462.15)	16,478.23 (6700.75)	16,437.95 (6259.03)	0.177	0.008 *	0.470
MDISTCE	7.2 (2.69)	6.86 (2.79)	6.84 (2.6)	0.177	0.008 *	0.470
MDISTMLCE	4.55 (2.14)	4.36 (2.09)	4.18 (2.02)	0.540	0.043 *	0.395
MDISTAPCE	4.6 (1.69)	4.34 (1.71)	4.48 (1.65)	0.916	0.096	0.414
RDISTCE	8.22 (3.11)	7.88 (3.15)	7.88 (2.98)	0.174	0.015 *	0.565
RDISTMLCE	5.67 (2.68)	5.5 (2.61)	5.32 (2.53)	0.350	0.033 *	0.559
RDISTAPCE	5.78 (2.13)	5.51 (2.14)	5.65 (2.09)	0.536	0.090	0.515
RANGECE	38.13 (15.11)	37.76 (15.42)	38.45 (15.66)	0.895	0.063	0.958
RANGEMLCE	31.05 (14.78)	31.14 (14.65)	30.89 (14.76)	0.658	0.016 *	0.985
RANGEAPCE	33.05 (13.42)	32.77 (13.53)	33.22 (14.29)	0.146	0.165	0.979
MVELCE	24.58 (12.71)	23.4 (11.36)	24.24 (15.06)	0.000 *	-	0.977
MVELMLCE	13.27 (6.89)	13.19 (6.82)	13.15 (8.9)	0.000 *	-	0.739
MVELAPCE	17.64 (10.32)	16.3 (8.69)	17.32 (10.98)	0.000 *	-	0.739
sRDCE	3.94 (1.61)	3.85 (1.5)	3.89 (1.49)	0.424	0.110	0.866
sAPMLCE	−1.05 (9.57)	−0.48 (13.63)	−0.7 (8.4)	0.006 *	-	0.298
AREACCCE	675.17 (529.15)	631.34 (506.34)	628.7 (433.01)	0.599	0.018 *	0.720
AREACECE	659.68 (539.88)	628.36 (497.18)	606.75 (428.13)	0.944	0.015 *	0.702
AREASWCE	63.54 (54.17)	60.29 (55.83)	60.86 (61.35)	0.000 *	-	0.850
MFREQCE	0.55 (0.25)	0.55 (0.17)	0.57 (0.23)	0.009 *	-	0.433
MFREQMLCE	0.55 (0.24)	0.56 (0.19)	0.56 (0.2)	0.315	0.114	0.870
MFREQAPCE	0.69 (0.36)	0.68 (0.28)	0.69 (0.33)	0.004 *	-	0.850
FDPDCE	1.78 (0.15)	1.76 (0.13)	1.76 (0.13)	0.991	0.083	0.483
FDCCCE	1.93 (0.2)	1.92 (0.15)	1.93 (0.18)	0.257	0.996	0.961
FDCECE	1.93 (0.2)	1.93 (0.15)	1.94 (0.18)	0.026 *	-	0.739
TPOWERRDCE	11.47 (9.05)	11.18 (9.65)	10.6 (8.64)	0.003 *	-	0.643
TPOWERMLCE	20.93 (23.84)	21.43 (28.32)	17.82 (21.91)	0.000 *	-	0.811
TPOWERAPCE	20.44 (18.44)	19.18 (23.21)	19.05 (17.26)	0.000 *	-	0.521
POWER50RDCE	0.52 (0.16)	0.55 (0.14)	0.54 (0.16)	0.021 *	-	0.452
POWER50MLCE	0.41 (0.14)	0.41 (0.13)	0.42 (0.12)	0.261	0.589	0.897
POWER50APCE	0.44 (0.15)	0.46 (0.14)	0.45 (0.15)	0.003 *	-	0.180
POWER95RDCE	1.98 (0.69)	2.04 (0.6)	2.06 (0.69)	0.185	0.035 *	0.429
POWER95MLCE	1.36 (0.54)	1.42 (0.46)	1.43 (0.42)	0.674	0.128	0.444
POWER95APCE	1.71 (0.76)	1.74 (0.65)	1.71 (0.69)	0.025 *	-	0.959
CFREQRDCE	0.97 (0.28)	1 (0.25)	1 (0.26)	0.037 *	-	0.211
CFREQMLCE	0.71 (0.23)	0.74 (0.19)	0.74 (0.18)	0.287	0.009 *	0.477
CFREQAPCE	0.84 (0.3)	0.87 (0.28)	0.84 (0.26)	0.007 *	-	0.811
FREQDRDCE	0.65 (0.04)	0.64 (0.03)	0.64 (0.04)	0.232	0.129	0.880
FREQDMLCE	0.61 (0.05)	0.63 (0.05)	0.62 (0.05)	0.558	0.126	0.149
FREQDAPCE	0.66 (0.06)	0.65 (0.05)	0.65 (0.05)	0.733	0.417	0.816

* *p*-value < 0.05.

**Table A6:** Statistical analysis of inter-rater reliability 78 CoP indices.

Cop Indices	ICC(2,1) (CI 95%)	Correlation Coefficient (*p*-Value)		
		Test 1–Test 2	Test 1–Test 3	Test 2–Test 3
RDOE	0.714 (0.555–0.874)	0.612 (0.000 *)	0.635 (0.000 *)	0.802 (0.000 *)
MDISTOE	0.714 (0.541–0.888)	0.612 (0.000 *)	0.635 (0.000 *)	0.802 (0.000 *)
MDISTMLOE	0.665 (0.473–0.858)	0.564 (0.000 *)	0.591 (0.000 *)	0.718 (0.000 *)
MDISTAPOE	0.549 (0.295–0.803)	0.419 (0.005 *)	0.486 (0.001 *)	0.633 (0.000 *)
RDISTOE	0.722 (0.558–0.886)	0.624 (0.000 *)	0.622 (0.000 *)	0.760 (0.000 *)
RDISTMLOE	0.631 (0.392–0.871)	0.577 (0.000 *)	0.574 (0.000 *)	0.698 (0.000 *)
RDISTAPOE	0.571 (0.324–0.817)	0.487 (0.001 *)	0.543 (0.000 *)	0.629 (0.000 *)
RANGEOE	0.603 (0.439–0.743)	0.646 (0.000 *)	0.551 (0.000 *)	0.544 (0.000 *)
RANGEMLOE	0.432 (0.118–0.746)	0.576 (0.000 *)	0.529 (0.000 *)	0.612 (0.000 *)
RANGEAPOE	0.686 (0.533–0.840)	0.599 (0.000 *)	0.649 (0.000 *)	0.551 (0.000 *)
MVELOE	0.789 (0.676–0.901)	0.782 (0.000 *)	0.724 (0.000 *)	0.719 (0.000 *)
MVELMLOE	0.739 (0.594–0.884)	0.645 (0.000 *)	0.720 (0.000 *)	0.597 (0.000 *)
MVELAPOE	0.789 (0.665–0.914)	0.844 (0.000 *)	0.815 (0.000 *)	0.866 (0.000 *)
sRDOE	0.639 (0.441–0.836)	0.643 (0.000 *)	0.594 (0.000 *)	0.645 (0.000 *)
sAPMLOE	0.047 (0.000–0.585)	0.072 (0.649)	0.333 (0.029 *)	0.132(0.400)
AREACCOE	0.717 (0.569–0.865)	0.623 (0.000 *)	0.598 (0.000 *)	0.720 (0.000 *)
AREACEOE	0.732 (0.583–0.880)	0.684 (0.000 *)	0.613 (0.000 *)	0.697 (0.000 *)
AREASWOE	0.768 (0.604–0.932)	0.730 (0.000 *)	0.666 (0.000 *)	0.638 (0.000 *)
MFREQOE	0.652 (0.500–0.778)	0.773 (0.000 *)	0.679 (0.000 *)	0.754 (0.000 *)
MFREQMLOE	0.656 (0.506–0.781)	0.643 (0.000 *)	0.681 (0.000 *)	0.697 (0.000 *)
MFREQAPOE	0.584 (0.417–0.728)	0.731 (0.000 *)	0.610 (0.000 *)	0.613 (0.000 *)
FDPDOE	0.629 (0.473–0.761)	0.722 (0.000 *)	0.612 (0.000 *)	0.591 (0.000 *)
FDCCOE	0.712 (0.576–0.820)	0.765 (0.000 *)	0.716 (0.000 *)	0.776 (0.000 *)
FDCEOE	0.745 (0.620–0.842)	0.783 (0.000 *)	0.740 (0.000 *)	0.825 (0.000 *)
TPOWERRDOE	0.663 (0.482–0.844)	0.777 (0.000 *)	0.701 (0.000 *)	0.613 (0.000 *)
TPOWERMLOE	0.655 (0.413–0.898)	0.671 (0.000 *)	0.696 (0.000 *)	0.711 (0.000 *)
TPOWERAPOE	0.706 (0.557–0.855)	0.740 (0.000 *)	0.767 (0.000 *)	0.709 (0.000 *)
POWER50RDOE	0.639 (0.484–0.768)	0.789 (0.000 *)	0.537 (0.000 *)	0.621 (0.000 *)
POWER50MLOE	0.516 (0.341–0.677)	0.691 (0.000 *)	0.434 (0.004 *)	0.486 (0.001 *)
POWER50APOE	0.556 (0.382–0.708)	0.602 (0.000 *)	0.609 (0.000 *)	0.570 (0.000 *)
POWER95RDOE	0.663 (0.513–0.786)	0.762 (0.000 *)	0.658 (0.000 *)	0.565 (0.000 *)
POWER95MLOE	0.728 (0.596–0.830)	0.799 (0.000 *)	0.763 (0.000 *)	0.661 (0.000 *)
POWER95APOE	0.659 (0.508–0.783)	0.688 (0.000 *)	0.697 (0.000 *)	0.642 (0.000 *)
CFREQRDOE	0.739 (0.582–0.895)	0.715 (0.000 *)	0.574 (0.000 *)	0.533 (0.000 *)
CFREQMLOE	0.702 (0.563–0.813)	0.830 (0.000 *)	0.665 (0.000 *)	0.624 (0.000 *)
CFREQAPOE	0.720 (0.588–0.825)	0.795 (0.000 *)	0.746 (0.000 *)	0.750 (0.000 *)
FREQDRDOE	0.482 (0.303–0.650)	0.500 (0.001 *)	0.572 (0.000 *)	0.389 (0.010 *)
FREQDMLOE	0.424 (0.241–0.602)	0.527 (0.000 *)	0.490 (0.001 *)	0.298 (0.053)
FREQDAPOE	0.608 (0.445–0.746)	0.575 (0.000 *)	0.653 (0.000 *)	0.672 (0.000 *)
RDCE	0.663 (0.514–0.786)	0.800 (0.000 *)	0.655 (0.000 *)	0.527 (0.000 *)
MDISTCE	0.663 (0.514–0.786)	0.800 (0.000 *)	0.655 (0.000 *)	0.527 (0.000 *)
MDISTMLCE	0.647 (0.494–0.774)	0.785 (0.000 *)	0.600 (0.000 *)	0.549 (0.000 *)
MDISTAPCE	0.670 (0.522–0.790)	0.717 (0.000 *)	0.729 (0.000 *)	0.559 (0.000 *)
RDISTCE	0.666 (0.517–0.788)	0.798 (0.000 *)	0.652 (0.000 *)	0.536 (0.000 *)
RDISTMLCE	0.644 (0.490 -0.772)	0.788 (0.000 *)	0.581 (0.000 *)	0.550 (0.000 *)
RDISTAPCE	0.673 (0.527–0.793)	0.738 (0.000 *)	0.714 (0.000 *)	0.560 (0.000 *)
RANGECE	0.609 (0.455–0.747)	0.725 (0.000 *)	0.594 (0.000 *)	0.496 (0.001 *)
RANGEMLCE	0.598 (0.432–0.740)	0.766 (0.000 *)	0.497 (0.001 *)	0.515 (0.000 *)
RANGEAPCE	0.580 (0.410–0.726)	0.643 (0.000 *)	0.619 (0.000 *)	0.467 (0.002 *)
MVELCE	0.724 (0.567–0.880)	0.734 (0.000 *)	0.800 (0.000 *)	0.706 (0.000 *)
MVELMLCE	0.684 (0.495–0.872)	0.749 (0.000 *)	0.762 (0.000 *)	0.764 (0.000 *)
MVELAPCE	0.752 (0.594–0.911)	0.788 (0.000 *)	0.838 (0.000 *)	0.696 (0.000 *)
sRDCE	0.635 (0.477–0.766)	0.752 (0.000 *)	0.613 (0.000 *)	0.519 (0.000 *)
sAPMLCE	0.276 (0.000–0.667)	0.276 (0.073)	0.174 (0.264)	0.213 (0.171)
AREACCCE	0.571 (0.401–0.720)	0.752 (0.000 *)	0.618 (0.000 *)	0.341 (0.025 *)
AREACECE	0.587 (0.420–0.731)	0.768 (0.000 *)	0.597 (0.000 *)	0.363 (0.017 *)
AREASWCE	0.581 (0.312–0.850)	0.829 (0.000 *)	0.709 (0.000 *)	0.698 (0.000 *)
MFREQCE	0.732 (0.584–0.880)	0.742 (0.000 *)	0.800 (0.000 *)	0.659 (0.000 *)
MFREQMLCE	0.606 (0.442–0.745)	0.633 (0.000 *)	0.557 (0.000 *)	0.662 (0.000 *)
MFREQAPCE	0.819 (0.711–0.927)	0.753 (0.000 *)	0.849 (0.000 *)	0.689 (0.000 *)
FDPDCE	0.751 (0.628–0.846)	0.834 (0.000 *)	0.702 (0.000 *)	0.741 (0.000 *)
FDCCCE	0.766 (0.648–0.856)	0.804 (0.000 *)	0.788 (0.000 *)	0.730 (0.000 *)
FDCECE	0.742 (0.595–0.890)	0.793 (0.000 *)	0.806 (0.000 *)	0.701 (0.000 *)
TPOWERRDCE	0.499 (0.202–0.796)	0.685 (0.000 *)	0.597 (0.000 *)	0.708 (0.000 *)
TPOWERMLCE	0.320 (0.000–0.716)	0.728 (0.000 *)	0.659 (0.000 *)	0.714 (0.000 *)
TPOWERAPCE	0.370 (0.000–0.745)	0.629 (0.000 *)	0.641 (0.000 *)	0.612 (0.000 *)
POWER50RDCE	0.511 (0.270–0.752)	0.419 (0.006 *)	0.558 (0.000 *)	0.598 (0.000 *)
POWER50MLCE	0.550 (0.376–0.704)	0.642 (0.000 *)	0.497 (0.001 *)	0.495 (0.001 *)
POWER50APCE	0.762 (0.622–0.902)	0.743 (0.000 *)	0.769 (0.000 *)	0.744 (0.000 *)
POWER95RDCE	0.774 (0.660–0.861)	0.860 (0.000 *)	0.777 (0.000 *)	0.697 (0.000 *)
POWER95MLCE	0.725 (0.594–0.828)	0.827 (0.000 *)	0.680 (0.000 *)	0.687 (0.000 *)
POWER95APCE	0.809 (0.701–0.918)	0.746 (0.000 *)	0.813 (0.000 *)	0.736 (0.000 *)
CFREQRDCE	0.746 (0.606–0.885)	0.707 (0.000 *)	0.756 (0.000 *)	0.683 (0.000 *)
CFREQMLCE	0.711 (0.575–0.819)	0.845 (0.000 *)	0.631 (0.000 *)	0.681 (0.000 *)
CFREQAPCE	0.825 (0.717–0.934)	0.743 (0.000 *)	0.824 (0.000 *)	0.753 (0.000 *)
FREQDRDCE	0.485 (0.303–0.654)	0.471 (0.005 *)	0.471 (0.005 *)	0.622 (0.000 *)
FREQDMLCE	0.606 (0.444–0.744)	0.713 (0.000 *)	0.489 (0.000 *)	0.623 (0.000 *)
FREQDAPCE	0.568 (0.397–0.717)	0.640 (0.000 *)	0.519 (0.000 *)	0.547 (0.000 *)

* *p*-value < 0.05
